# Long‐term survival outcome with tyrosine kinase inhibitors and surgical intervention in patients with metastatic or recurrent gastrointestinal stromal tumors: A 14‐year, single‐center experience

**DOI:** 10.1002/cam4.1994

**Published:** 2019-01-28

**Authors:** Jwa Hoon Kim, Min‐Hee Ryu, Changhoon Yoo, Heejung Chae, Hana Na, Moyoul Beck, Beom Su Kim, Moon‐Won Yoo, Jeong Hwan Yook, Byung Sik Kim, Ki‐Hun Kim, Chan Wook Kim, Yoon‐Koo Kang

**Affiliations:** ^1^ Department of Oncology Asan Medical Center, University of Ulsan College of Medicine Seoul Korea; ^2^ Department of Surgery, Asan Medical Center University of Ulsan College of Medicine Seoul Korea

**Keywords:** gastrointestinal stromal tumor, imatinib mesylate, survival

## Abstract

The long‐term effects of tyrosine kinase inhibitors (TKIs), including imatinib, and surgical intervention on advanced gastrointestinal stromal tumor (GIST) were evaluated. All 379 patients had metastatic or recurrent GIST and started 400 mg/d imatinib at the Asan Medical Center in periods 1 and 2 [2001‐2007 (33.2%) and 2008‐2014 (66.8%), respectively]. Men constituted 60.4%; median patient age and tumor size at the initiation of imatinib were 58.6 (14.6‐85.5) years and 51 (0‐324) mm, respectively, without differences between periods except for older age and less preimatinib surgery in period 2. Response and disease control rates with imatinib in measurable GIST were 63.1% and 94.3%, respectively, without differences between periods. More patients in period 2 underwent surgical resection for TKI‐responsive diseases within the first 2 years (24.9%, *P = *0.006). With a median follow‐up of 6.1 years (2.5‐16.0) in survivors, median progression‐free survival (PFS) was 5.4 years [95% confidence interval (CI), 4.0‐6.9]. Subsequent sunitinib (*P = *0.066) and regorafenib (*P = *0.003) were more commonly administered in period 2. Median overall survival (OS) was 8.8 years (95% CI, 7.8‐9.7). PFS with imatinib (*P = *0.002) and OS (*P = *0.019) were significantly longer in period 2. Young age, smaller tumor size at the initiation of imatinib, KIT exon 11 mutation, surgical intervention, and period 2 were favorable factors for PFS and OS. Patients with advanced GIST showed better prognosis with the optimal use of imatinib, along with active surgical intervention and more common use of subsequent TKIs in period 2.

## 1 INTRODUCTION

Gastrointestinal stromal tumors (GISTs) are the most common mesenchymal tumors of the gastrointestinal tract. The development of imatinib, a KIT‐ or platelet‐derived growth factor receptor A (PDGFRA)‐targeting tyrosine kinase inhibitor (TKI), has revolutionized the treatment and survival outcomes of advanced GISTs.^1^, ^2^, ^3^ The first multicenter phase 2 trial (B2222 trial)^4^, ^5^ and the Southwest Oncology Group (SWOG) and European Organisation for Research and Treatment of Cancer (EORTC) phase III trials^1^, ^2^, ^6^ demonstrated significant improvements with imatinib in the objective response rate (ORR), median progression‐free survival (PFS), and median overall survival (OS) (ORR, 68, 45, and 51%; median PFS, 1.7, 1.5, and 1.7 years; and median OS, 4.8, 4.3, and 3.9 years, respectively).

Most patients with advanced GISTs eventually develop clinical resistance to imatinib. Dose escalation of imatinib up to 800 mg/d or sunitinib as the new generation KIT‐ or PDGFRA‐targeting TKI are currently used to overcome clinical resistance to 400 mg/d imatinib since the mid‐2000.^7^, ^8^ After the failure of both imatinib and sunitinib, regorafenib, which is also a novel oral multikinase inhibitor targeting KIT or PDGFRA, was approved for the third‐line therapy in 2013^9^, ^10^ and a few investigational therapies^11^, ^12^, ^13^ have been evaluated based on the limited treatment options. If regorafenib is unavailable or ineffective, resumption of imatinib is recommended instead of the discontinuation of TKI treatment.^14^ Moreover, beginning in 2000, surgical resection of residual lesions after disease control with imatinib has been shown beneficial, and it has been considered or recommended in the guidelines since the mid‐2000s to prevent or delay the emergence of secondary resistance in patients with metastatic or recurrent GISTs.^15^

Similarly, the National Comprehensive Cancer Network (NCCN),^16^ European Society of Medical Oncology (ESMO),^17^ and Asian Consensus^18^ guidelines for the treatment and management of GISTs have been published. Initial treatment with imatinib at 800 mg/d has been recommended for patients with KIT exon 9 mutation in Western countries; whereas, an initial higher dose of imatinib has not yet been used as the standard in Asian patients with a similar genotype. Although Asian patients with KIT exon 9 mutation could also benefit from treatment with an initial higher dose of imatinib, there have been no large prospective studies in Asian countries, and there are concerns about its feasibility and safety.^18^, ^19^ The feasibility and efficacy of high‐dose imatinib as initial dose are currently being explored in Asian patients with KIT exon 9 mutation (KENEDI study: NCT01541709).

Data are lacking on the long‐term outcome of metastatic or recurrent GISTs associated with these treatment advances. Therefore, we aimed to investigate their clinical impact on survival by comparing survival outcomes between early and late periods, and identify the prognostic factors over the past 14 years at a single institution.

## 2 MATERIAL AND METHODS

### 2.1 Patients

We reviewed the records of all patients who were treated for histologically confirmed advanced GISTs and registered in a prospective database at the Asan Medical Center (AMC, Seoul, Korea) between January 2001 and December 2014. Patients were treated with 400 mg/d imatinib as the first‐line therapy for metastatic (initially presenting metastatic disease) or recurrent (recurrence of either local or distant tumors or both after previous surgical resection) GISTs. Patients who were initiated on 400 mg/d imatinib at AMC or another hospital but were transferred to AMC within 3 months of initiation of imatinib were included, which left 379 eligible patients. They were classified into period 1 (2001‐2007) and period 2 (2008‐2014) according to the initiation date of imatinib. The study protocol was approved by the Institutional Review Board of the AMC, and patient medical records were reviewed.

### 2.2 Treatment and evaluation

Our standard protocol for administering imatinib and subsequent new generation KIT‐ or PDGFRA‐ targeting TKIs and performing surgical intervention during TKIs met the NCCN,^16^ ESMO,^17^ or Asian consensus^18^ guidelines. All patients with metastatic or recurrent GISTs were administered imatinib at an initial dose of 400 mg daily until progressive disease (PD) or intolerable toxicities occurred, and the same dose was administered to patients with KIT exon 9 mutation.

Doses were carefully modified according to toxicities and imatinib plasma concentration to maintain sufficient imatinib treatment for achieving an optimal outcome.^20^, ^21^ Toxicities with laboratory tests were examined in every outpatient clinic and unscheduled visits in the emergency room were notified to our GIST team. Proper management of toxicities also enabled the continuation of imatinib administration. Erythropoietin was administered to patients who had significant anemia (Hb <10) without iron or vitamin B12 deficiency, and imatinib dose intensity could be maintained by simultaneously administering systemic steroid in patients with severe skin rashes that required further intervention.^23^ Imatinib plasma concentration monitoring was routinely used to guide dose modification and successfully manage toxicity or reaction to imatinib. These events were sometimes mistaken for disease progression, and careful monitoring prevented unnecessary imatinib interruption.^24^

The objective response of GIST to 400 mg/d imatinib was evaluated according to the Response Evaluation Criteria in Solid Tumors (RECIST) version 1.0.^25^ Consistent with the SWOG criteria, an increase in the sum of the longest diameters of target lesions alone was not regarded as PD if it was accompanied by definite cystic changes in the tumor, suggesting necrosis.^26^ Focal PD (FPD) was defined as progression of single or two preexisting sites, which was either an increase in size or the development of a new enhancing focus enclosed within a nonenhancing tumor mass^27^ and single new lesion, despite other remaining lesions were controlled, was also regarded as FPD. Generalized PD (GPD) was defined as an increase in tumor size, tumor density, and heterogeneous enhancing pattern in more than two tumor masses.^28^ Furthermore, 18‐fluorodeoxyglucose (FDG)‐positron emission tomography (PET) was performed in case when computed tomography (CT) scans alone were not definitive for response evaluation.

In patients with disease progression following 400 mg/d imatinib, the dose was escalated to 800 mg/d if patients were able to tolerate this high dose.^7^ For patients unable to tolerate 800 mg/d imatinib, the dose was reduced to 600 mg. Sunitinib, which has been available since 2006, was administered after failure of higher dose of imatinib (600 or 800 mg/d).^8^ A few patients with severe toxicity to imatinib were administered sunitinib after disease progression with 400 mg/d imatinib. The dose of sunitinib was 50 mg/d for 4 weeks, followed by a 2‐week resting period (4/2 schedule) in the early days of sunitinib introduction,^29^ and 37.5 mg/d sunitinib was later administered with equivalent effects.^30^ For patients who were unable to tolerate 37.5 mg/d sunitinib, the dose was reduced to 25 mg/d or 25 mg for 2 weeks, followed by a 1‐week resting period (2/1 schedule). Subsequently, patients were treated with regorafenib, which has been available since 2013,^9^ and imatinib was resumed after regorafenib failure or its unavailability.^14^ A few investigational therapies [eg, dovitinib,^11^ nilotinib,^13^ and sorafenib^12^] or supportive care strategies were provided to patients with metastatic or recurrent GISTs after failure of both imatinib and sunitinib, depending on physician's decision.

Surgical resection in responsive disease (RD) with imatinib has been considered or recommended since the mid‐2000s after disease control [partial response (PR) or stable disease (SD)] for at least 6 months following imatinib. Surgical resection in FPD with TKIs was occasionally performed after multidisciplinary discussion. In GPD with TKIs or initial metastatic GISTs, surgery was not recommended except for some patients. Palliative surgery was performed only in patients with relevant symptoms such as gastrointestinal compression or obstruction and bleeding due to the tumor mass. Furthermore, some patients with initial metastatic GISTs, before the era of imatinib or transferred from other hospitals, also underwent initial cytoreductive surgery.

### 2.3 Genotype

KIT exons 9, 11, 13, and 17, and PDGFRA exons 12 and 18 were amplified using polymerase chain reaction (PCR) and Sanger sequencing at the time of diagnosis according to previously described procedures.^19^, ^22^ The wild type was defined as no mutation in KIT exons 9, 11, 13, and 17 and PDGFRA exons 12 and 18.

### 2.4 Statistical analysis

Differences in the baseline characteristics of patients in the two periods were compared using the Chi‐square and Fisher's exact tests for categorical variables and the *t*‐test for continuous variables. The ORR between the two periods was compared using the Chi‐square, and prognostic factor analysis for ORR was analyzed using a logistic regression model. OS was calculated from the date of initiation of 400 mg/d imatinib to the date of death resulting from any cause. PFS was calculated from the date of initiation of 400 mg/d imatinib to the date of PD with 400 mg/d imatinib or death resulting from any cause. PD with 400 mg/d imatinib was determined only when tumors were not controlled with 400 mg/d imatinib regardless of any involvement of surgical intervention. Specifically, patients who underwent surgical resection in RD with imatinib were not censored at the time of surgery and were continuously followed up until PD with 400 mg/d imatinib. Furthermore, patients with FPD with 400 mg/d imatinib were also considered as PD regardless of whether surgical intervention was performed.

Survival rates were estimated using the Kaplan–Meier method, and the log‐rank test was used to compare differences between the curves. Prognostic factors for PFS and OS were analyzed using Cox proportional hazard regression model. The multivariate analysis included factors considered significant (*P < *0.1) in the univariate analysis, and adjusted hazard ratios (HR) with 95% confidence intervals (CIs) were calculated. A two‐sided *P* < 0.05 was considered significant, and all statistical analyses were performed using the statistical package for the social sciences (SPSS) 18.0 software package (IBM SPSS Statistics, Chicago, IL, USA).

## 3 RESULTS

### 3.1 Patient characteristics

Baseline characteristics are summarized and compared between the periods in Table 1. A total of 379 patients with metastatic or recurrent GISTs were classified into periods 1 and 2, consisting of 126 (33.2%) and 253 (66.8%) patients, respectively. The median age was 58.6 years (range, 14.6‐85.5 years), and 60.4% were men. There were no significant differences between the two periods except for older age in period 2 and higher proportion of preimatinib surgery in period 1.

**Table 1:** Baseline characteristics of patients who were initiated on 400 mg/d imatinib as the first‐line treatment

	All periods (n = 379)	Period 1 (n = 126)	Period 2 (n = 253)	*P*‐value
Median age (range), years	58.6 (14.6‐85.5)	56.1 (31.2‐85.5)	59.0 (14.6‐82.9)	0.018
Age >60 y	170 (44.9%)	53 (42.1%)	117 (46.2%)	0.441
Sex (male)	229 (60.4%)	83 (65.9%)	146 (57.7%)	0.126
Primary tumor sites, n (%)				0.866
Stomach	154 (40.6)	49 (38.9)	105 (41.5)	
Small intestine	179 (47.2)	60 (47.6)	119 (47.0)	
Colon and rectum	22 (5.8)	9 (7.1)	13 (5.1)	
Others^a^	24 (6.3)	8 (6.3)	16 (6.3)	
Disease status, n (%)				0.624
Initial metastatic GISTs	151 (39.8)	48 (38.1)	103 (40.7)	
Recurrent GISTs	228 (60.2)	78 (61.9)	150 (59.3)	
Diameter of the largest lesions^b^				
Median size at the presentation of advanced GISTs (mm, range)	58 (6‐324)	56 (7‐170)	59 (6‐324)	0.158
Median size at the start of imatinib treatment (mm, range)	51 (0‐324)	50 (0‐170)	53 (0‐324)	0.276
Preimatinib surgery	92 (24.2%)	40 (31.7%)	52 (20.6%)	0.017
No evaluable lesions after initial cytoreductive surgery	28 (7.4%)	8 (6.3%)	20 (7.9%)	0.585
Sites of metastasis				
Liver	212 (55.9%)	73 (57.9%)	139 (54.9%)	0.580
Lung	1 (0.3%)	1 (0.8%)	0 (0.0%)	0.156
Peritoneum	166 (43.8%)	59 (46.8%)	107 (42.3%)	0.402
Liver and Peritoneum	60 (15.8%)	26 (20.6%)	34 (13.4%)	0.071
Genotype of primary tumor^c^				0.821
KIT exon 11 mutation	261 (72.5%)	80 (70.2%)	181 (73.6%)	
KIT exon 9 mutation	39 (10.8%)	12 (10.5%)	27 (11.0%)	
Wild‐type^d^	29 (8.1%)	10 (8.8%)	19 (7.7%)	
Others^e^	18 (5.0%)	6 (5.3%)	12 (4.9%)	
Undetermined	13 (3.6%)	6 (5.3%)	7 (2.8%)	

GISTs, gastrointestinal stromal tumors; PDGFRA, platelet‐derived growth factor receptor A.

a Esophagus (n = 3), omentum and peritoneum (n = 18), and retroperitoneum (n = 3).

b No available data (n = 3): no CT scan.

c Available specimen analyzed (n = 360).

d No mutation in KIT exon 9, 11, 13, and 17 and PDGFRA exon 12 and 18.

e PDGFRA 12 or 18 mutation (n = 10), KIT exon 13 mutation (n = 1), KIT exon 17 mutation (n = 3), and double mutation in KIT or PDGFRA exon (n = 4); KIT exon 9 and 11 (n = 3), and KIT exon 9 and PDGFRA exon 12 (n = 1).

Among 360 patients with available tumor specimens that were examined before imatinib treatment, 318 (88.3%) had KIT or PDGFRA mutation and the wild type was observed in 29 patients (8.1%). The most frequent KIT or PDGFRA mutation was the KIT exon 11 mutation (n = 261, 72.5%), followed by KIT exon 9 mutation (n = 39, 10.8%), PDGFRA exon 12 or 18 mutation (n = 10, 2.8%; including PDGFRA D842V mutation, n = 3, 0.8%), KIT exon 17 mutation (n = 3, 0.8%), KIT exon 13 mutation (n = 1, 0.3%), and double mutations in KIT or PDGFRA exon (n = 4, 1.1%).

### 3.2 Clinical response to 400 mg/d imatinib and correlation of tumor genotype

The antitumor responses with 400 mg/d imatinib were investigated in 331 patients with measurable disease, and the ORR and disease control rate (DCR) were compared between the two periods. Overall, 24 (7.3%), 185 (55.9%), 103 (31.1%), and 12 (3.6%) patients showed complete response, PR, SD, and PD, respectively. Additionally, 7 (2.1%) patients were not assessable; of these, four were lost to follow‐up, two had no earlier CT scan, and one had a combination of other malignancies.

ORR and DCR with imatinib were 63.1% and 94.3%, respectively, in all patients with measurable GISTs. There was no significant difference between the two periods. Univariable prognostic factor analysis of ORR identified KIT mutation status as significant, where patients with KIT exon 11 mutation had significantly higher odds of achieving a response than those with KIT exon 9 mutation [odds ratio (OR), 5.10; 95% CI, 2.39‐10.87; *P < *0.001] and the wild‐type mutation (OR, 3.11; 95% CI, 1.36‐7.08; *P = *0.007).

### 3.3 Subsequent treatments after failure of 400 mg/d imatinib and surgical resection of residual lesions during TKI treatment

Table 2 summarizes and compares the two periods for additional treatments in patients who were initiated on 400 mg/d imatinib. 400 mg/d imatinib was discontinued at AMC because of disease progression (n = 184, 48.5%), loss to follow‐up or transfer to other hospitals (n = 34, 8.9%), toxicity (n = 11, 2.9%), prolonged absence of gross lesions on CT scan after imatinib or surgical intervention (n = 9, 2.4%), and patient refusal (n = 3, 0.8%). Among 129 patients treated with the higher dose of imatinib (600 or 800 mg/d), 13 patients (10.1%) were on imatinib treatment, and 116 patients (89.9%) discontinued imatinib treatment because of disease progression (n = 103, 79.8%), loss to follow‐up or transfer to other hospitals (n = 6, 4.7%), toxicity (n = 4, 3.2%), and refusal (n = 3, 2.3%). Sunitinib was administered after failure of the higher dose of imatinib or 400 mg/d imatinib to all 122 patients. Dose schedules were 50 mg/d (4/2‐week schedule; n = 20, 16.4%) and 37.5 mg/d (n = 61, 50%). If patients were not able to tolerate initial 50 mg/d (4/2‐week schedule) or 37.5 mg/d sunitinib, dose was reduced to 25 mg/d in continuous or 2/1‐week schedule (n = 41, 33.6%).

**Table 2:** Additional treatments by time periods in patients who were initiated on 400 mg/d imatinib as the first‐line treatment

Subsequent treatments after progressive disease (PD) with 400 mg/d imatinib				
Patients showed PD with 400 mg/d imatinib	All periods (n = 184)	Period 1 (n = 87)	Period 2 (n = 97)	*P*‐value
Second‐line treatment	158 (85.9%)	72 (82.8%)	86 (88.7%)	0.154
Dose of imatinib escalated^a^	129 (70.1%)	55 (63.2%)	74 (76.3%)	
Sunitinib administered	29 (15.8%)	17 (19.5%)	12 (12.4%)	
Patients showed PD with dose escalation of imatinib	All periods (n = 103)	Period 1 (n = 44)	Period 2 (n = 59)	*P*‐value
Sunitinib administered	93 (90.3%)	37 (84.1%)	56 (94.9%)	0.066
Patients showed PD with both imatinib and sunitinib	All periods (n = 94)	Period 1 (n = 45)	Period 2 (n = 49)	*P*‐value
Regorafenib administered	13 (13.8%)	4 (8.9%)	9 (18.4%)	0.003
Resumption of imatinib or investigational therapies^b^ administered	56 (59.6%)	24 (53.3%)	32 (65.3%)	0.046
Surgical resection of residual lesions during TKIs treatment				
All patients initiated 400 mg/d imatinib	All periods (n = 379)	Period 1 (n = 126)	Period 2 (n = 253)	*P*‐value
Surgical resection in RD with TKIs within the first 2 y of starting TKIs	79 (20.8%)	16 (12.7%)	63 (24.9%)	0.006

Abbreviations: PD, progressive disease; RD, responsive disease; TKI, tyrosine kinase inhibitors.

a 600 or 800 mg/d imatinib.

b Dovitinib, nilotinib, sorafenib.

Among the 379 patients, 158 (41.7%) underwent surgical resection of residual lesions during TKI treatment. Of the 158 patients, 109 (28.8%), 31 (8.2%), and 18 (4.7%) underwent surgical resection in RD, FPD, and GPD, respectively. Surgical resection in RD was performed during imatinib or sunitinib treatment (n = 103 or 6, respectively). Most patients underwent surgery within the first 2 years of starting 400 mg/d palliative TKIs, and more surgeries were performed in period 2 than in period 1 (12.7% vs 24.9%, *P = *0.006). There was a higher trend of increasing frequency of sunitinib administration after the failure of dose escalation of imatinib in period 2 than in period 1 (*P = *0.066). Regorafenib and resumption of imatinib or investigational therapies were more commonly administered to patients after failure of both imatinib and sunitinib in period 2 than in period 1 (*P = *0.003 and *P = *0.046, respectively).

### 3.4 PFS and OS

**Figure 1:**
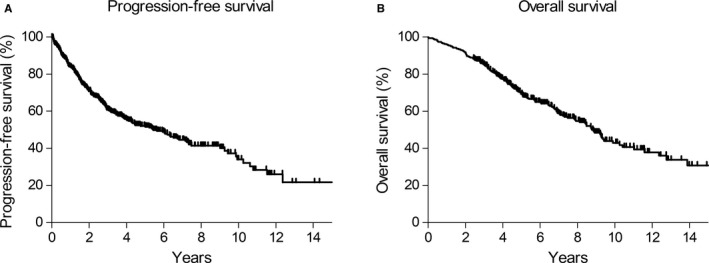
(A) Progression‐free survival (PFS) with 400 mg/d imatinib and (B) overall survival (OS) in all 379 patients with metastatic or recurrent gastrointestinal stromal tumors who were initiated on 400 mg/d imatinib as the first‐line treatment (median PFS, 5.4 y and median OS, 8.8 y)

A median follow‐up of 6.1 years (range, 2.5‐16.0 years) in survivors was accompanied by disease progression with 400 mg/d imatinib in 184 (48.5%), and 159 (42.0%) died because of GISTs. The median PFS and OS were 5.4 years (95% CI, 4.0‐6.9) and 8.8 years (95% CI, 7.8‐9.7), respectively (Figure 1A,B). The estimated 8‐ and 10‐year survival rates were as follows: 8‐year PFS and OS rate was 41% and 54.7%, and 10‐year PFS and OS rate was 34% and 42.9%, respectively.

**Figure 2:**
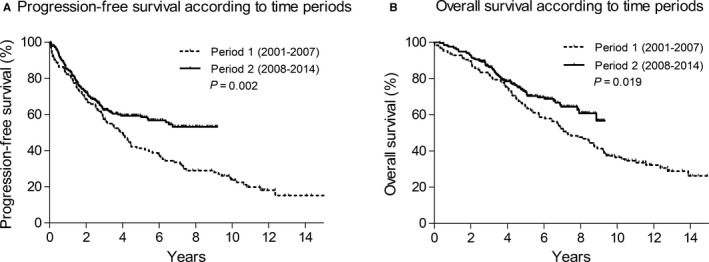
(A) Progression‐free survival (PFS) with 400 mg/d imatinib and (B) overall survival (OS) according to time periods in patients with metastatic or recurrent gastrointestinal stromal tumors who were initiated on 400 mg/d imatinib as the first‐line treatment. (A) Median PFS with 400 mg/d imatinib and (B) median OS in period 2 were significantly greater than those in period 1 (median PFS, not reached vs 3.9 years, *P* = 0.002; median OS, not reached vs 7.2 y, *P* = 0.019, respectively)

Comparing period 2 with 1, PFS with 400 mg/d imatinib was significantly longer in period 2 than it was in period 1 (not reached vs 3.9 years, *P = *0.002, Figure 2A). The median OS in period 2 was significantly greater than it was in period 1 (not reached vs 7.2 years, *P = *0.019, Figure 2B). Table 3 summarizes the results of the prognostic factor analysis of PFS and OS. In the univariate and multivariate analyses of both PFS with 400 mg/d imatinib and OS, independent favorable factors were age <60 years, smaller tumor size at the initiation of imatinib, the presence of KIT exon 11 mutation, disease control with 400 mg/d imatinib, surgical resection in RD with TKIs, and treatment in period 2.

**Table 3:** Univariate and multivariate analyses of prognostic factors for progression‐free survival (PFS) and overall survival (OS)

Variables	Progression‐free survival (PFS)				Overall survival (OS)			
	Univariate analysis		Multivariate analysis		Univariate analysis		Multivariate analysis	
	HR (95% CI)	*P*‐value	HR (95% CI)	*P*‐value	HR (95% CI)	*P*‐value	HR (95% CI)	*P*‐value
Age >60 vs <60 y	1.56 (1.17‐2.07)	0.002	1.47 (1.09‐2.00)	0.013	1.94 (1.41‐2.65)	<0.001	1.71 (1.22‐2.41)	0.002
Sex, M vs F	1.23 (0.92‐1.65)	0.159			1.35 (0.97‐1.86)	0.073		
Disease status^a^	1.28 (0.96‐1.70)	0.084			1.42 (1.04‐1.94)	0.028		
Median diameter of the largest lesions^b^	1.04 (1.01‐1.07)	0.003	1.06 (1.03‐1.09)	<0.001	1.07 (1.04‐1.10)	<0.001	1.08 (1.05‐1.12)	<0.001
Genotype								
KIT Exon 11	1.00		1.00		1.00		1.00	
Non‐KIT Exon 11	1.78 (1.29‐2.45)	<0.001	2.05 (1.44‐2.91)	<0.001	1.51 (1.06‐2.16)	0.024	2.15 (1.48‐3.14)	<0.001
Wild‐type	1.24 (0.73‐2.13)	0.426			1.15 (0.63‐2.10)	0.641		
Others	1.23 (0.65‐2.52)	0.477			1.78 (0.93‐3.41)	0.083		
KIT Exon 9	2.84 (1.89‐4.27)	<0.001			1.68 (1.05‐2.70)	0.030		
Disease control^c^, Y vs N	0.02 (0.01‐0.03)	<0.001	0.01 (0.01‐0.03)	<0.001	0.39 (0.19‐0.76)	0.006	0.41 (0.20‐0.85)	0.017
Dose reduction of imatinib for at least 3 mo, Y vs N	0.68 (0.43‐1.07)	0.094			1.12 (0.73‐1.73)	0.608		
Preimatinib surgery, Y vs N	0.73 (0.51‐1.03)	0.073	0.89 (0.57‐1.39)	0.608	0.70 (0.47‐1.02)	0.065	0.81 (0.50‐1.33)	0.404
No evaluable lesions on CT scan after initial cytoreductive surgery, Y vs N	0.66 (0.35‐1.24)	0.194			0.56 (0.26‐1.20)	0.137		
Surgical resection in RD with TKIs, Y vs N	0.58 (0.41‐0.82)	0.002	0.48 (0.33‐0.69)	<0.001	0.53 (0.35‐0.79)	0.002	0.40 (0.26‐0.61)	<0.001
Periods								
Period 1	1.00		1.00		1.00		1.00	
Period 2	0.63 (0.47‐0.84)	0.002	0.72 (0.53‐0.97)	0.033	0.67 (0.48‐0.94)	0.020	0.75 (0.53‐1.06)	0.062

Abbreviations: CI, confidence interval; GIST, gastrointestinal stromal tumor; HR, hazard ratio; RD, responsive disease; TKI, tyrosine kinase inhibitors.

a Initial metastatic GISTs compared with recurrent GISTs.

b Hazard ratio per 10 mm increase.

c Complete response, partial response, and stable disease with 400 mg/d imatinib.

### 3.5 PFS and OS in patient subgroups

Patients were classified according to favorable factors for survival. In patients with KIT exon 11 mutation, the median PFS and OS was 6.7 years (95% CI, 4.5‐9.0) and 9.3 years (95% CI, 8.1‐10.4), respectively. In patients aged <60 years, the median PFS and OS was 7.2 years (95% CI, 5.0‐9.3) and 11.0 years (95% CI, 7.8‐14.2), respectively. In patients with median diameters of the largest lesions <50 mm, the median PFS and OS was 7.3 years (95% CI, 4.9‐9.7) and 11.6 years (95% CI, 8.9‐14.3), respectively.

## 4 DISCUSSION

In our study, the survival outcome in patients with metastatic or recurrent GISTs who were initiated on 400 mg/d imatinib was better than that reported by previous large clinical trials in terms of median PFS and OS. The early imatinib trials (B2222 phase II and SWOG and EORTC phase III trials) demonstrated median PFS and OS values of approximately 1‐2 and 4‐5 years, respectively.^2^, ^4^, ^5^, ^6^, ^31^ In contrast, the median PFS and OS of our study is at least 3 years longer than those reported in the trials (median PFS and OS, 5.4 and 8.8 years, respectively). Within the population in AMC, patients in period 2 show better improvements in median PFS and OS than those in period 1 (*P = *0.002 and *P = *0.019, respectively). Surgical resection in RD with TKIs within the first 2 years of starting TKIs was more commonly performed in period 2 (*P = *0.006) than in period 1, and subsequent TKIs and resumption of imatinib or investigational therapies in PD with imatinib, sunitinib, or both were more commonly administered in period 2 (sunitinib, *P = *0.066; regorafenib, *P = *0.003; and resumption of imatinib or investigational therapies, *P = *0.046) than in period 1. Younger age, smaller tumor size at the initiation of imatinib, the presence of exon 11 mutation, and surgical resection in RD with TKIs were also favorable prognostic factors for imatinib treatment in metastatic or recurrent GISTs.

Comparisons between early imatinib trials,^1^, ^2^, ^4^ recent studies^32^, ^33^ (registered until 2010 or 2013), and the present study are summarized in Table 4. Although baseline demographic features and KIT or PDGFRA exon mutation were similar between studies, there were differences in tumor burden and surgical intervention. The size of the largest tumor tends to be smaller in AMC and recent studies except for Taiwan than it was in early imatinib trials, and lower tumor burden may be one of the possible explanations for the survival difference. Active surgical resection in RD with TKIs was recently performed in 21.9% of Polish patients and in 28.8% of our patients compared with 3.7% of the EORTC trial,^6^ which was independently associated with better survival. Furthermore, tumor density as well as tumor size was comprehensively considered to evaluate the tumor response according to RECIST along with SWOG criteria. Also, we occasionally performed FDG‐PET scan to understand the metabolic response, which could indicate the portion of viable tumor cells. These approaches enabled our team to continue imatinib without unnecessary interruption. Some discontinuation of imatinib in previous trials may have resulted from the underestimated responses of GISTs before the maximum effect appeared in slow responders, which may have influenced the median PFS.

**Table 4:** Comparison between early imatinib trials, recent studies, and present study

	B2222	SWOG	EORTC	Norway	Taiwan	Poland	AMC
Patients	2000‐2001 (n = 147)	2001‐2002 (n = 694)	2001‐2002 (n = 946)	1995‐2013 (n = 115)	2001‐2010 (n = 171)	2001‐2010 (n = 430)	2001‐2014 (n = 379)
Median diameter of the largest lesion (mm, %)	—	—	80	<50 (57), >50 (43)	100	73	51
ORR (%) with 400 mg/d IM	68	45	51	—	57.3	—	63.1
KIT exon 11 compared with non‐KIT exon 11	Significantly higher OR	Significantly higher OR	Significantly higher OR	—	Significantly higher OR	—	Significantly higher OR
DCR (%) with IM 400 mg daily	82.2	70	84.8	—	87.1	—	94.3
Surgical resection in RD with TKIs (%)	—	—	3.7	8	—	21.9	28.8
Median PFS (years) with 400 mg/d IM	1.7	1.5	1.7	—	3.1	3.1	5.4
Median OS (years)	4.8	4.3	3.9	6.9	5.9	5.8	8.8

Abbreviations: DCR, disease control rate; IM, imatinib; OR, odd ratio; ORR, objective response rate; OS, overall survival; PFS, progression‐free survival; PS, performance status; RD, responsive disease; TKI, tyrosine kinase inhibitor.

In our study, the progression of GIST in period 2 is more delayed, and patients in period 2 live longer than those in period 1. Active combination of surgical resection in RD with TKIs was more commonly performed in period 2 and is responsible for better survival than in period 1. Surgical resection of resistant or unresponsive clones could contribute to prolonging the disease control period, and reducing the tumor burden may decrease the risk of secondary resistance.^35^ We previously demonstrated that surgical resection of residual lesions after disease control with imatinib significantly improved the median PFS (HR, 2.33; 95% CI, 1.03‐5.24; *P = *0.041) and median OS (HR, 5.46; 95% CI, 1.46‐20.41; *P = *0.012).^15^ Several retrospective studies also advocated that the addition of surgical resection at the maximal clinical response of GIST to imatinib (PR or SD) during imatinib treatment may be associated with survival benefits.^36^, ^37^ Furthermore, more widespread use of subsequent TKIs and the resumption of imatinib or investigational therapies may have led to survival benefits for patients in period 2 after PD treated with imatinib.

With long‐term follow‐up duration, estimated 8‐ and 10‐year OS rates were 54.7% and 42.9%, respectively. These are also numerically higher than the 8‐ and 10‐year OS rate of 31% and 23% in the SWOG trial and 10‐year OS rate of 19.4% in the EORTC trial. Consistent with early imatinib trials and recent studies,^2^, ^5^, ^6^, ^31^, ^32^, ^33^ the long‐term survivors of our study are characterized by younger age, smaller tumor size at the initiation of imatinib, and the presence of exon 11 mutation. Among 126 patients in period 1 with follow‐up >10 years, 42 patients (33.3%) were alive for more than 10 years at the time of the analysis. Survivors over 10 years, approximately one third in period 1, had smaller tumor sizes at the initiation of imatinib, younger age, and underwent more surgical resection in RD with TKIs compared with remaining patients in period 1 (Table S1). In patient subgroups with each favorable factor, median PFS and OS are approximately 7 and 9‐11 years, respectively, and in the present study, these are better than values in overall patients. Notably, some patients with advanced GISTs treated with 400 mg/d imatinib alone show favorable survival without disease progression, and there are patients with advanced GISTs who live more than 10 years.

The strength of this study is that our results show the advancements in GIST therapy by comparing the survival outcomes between two periods in a relatively large cohort and applying consistent treatment strategies at a single center. Our study reveals the clinical impact of second‐ or later‐line treatments wherein the data are limited in previous studies. Moreover, the results highlight the clinical significance of surgical intervention by revealing its independent role in both PFS and OS in advanced GISTs with imatinib treatment.

## 5 CONCLUSION

The current data obtained from the AMC provides the long‐term outcome of patients with advanced GISTs treated with tyrosine kinase inhibitors including imatinib, followed by active surgical intervention and investigational therapies. Survival outcomes over 14 years in AMC suggest that multimodality management with thorough experience in GIST in line with current guidelines would lead to better survival outcomes compared to early trials when TKIs were first introduced.
