# Supplementary material for: Long‐term survival outcome with tyrosine kinase inhibitors and surgical intervention in patients with metastatic or recurrent gastrointestinal stromal tumors: A 14‐year, single‐center experience
Source: Cancer Med. 2019 Jan 28;8(3):1034–43. doi: 10.1002/cam4.1994 (PMC6434201; doi:10.1002/cam4.1994)
Supplement: Supplementary file 1 [file CAM4-8-1034-s001.doc]

**Supporting information**

Supplement Table 1. Baseline characteristics between survivors over 10 years and remaining patients in period 1

|  | Survivors over 10 years (n = 42) | Remaining patients in period 1 (n = 84) | *p*-value |
| --- | --- | --- | --- |
| Median age, years (range) | 52.4 (32.6–69.5) | 57.7 (31.2–85.5) | 0.018 |
| Young age < 60 years (%) | 30 (71.4) | 43 (51.2) | 0.030 |
| Sex (male, %) | 22 (52.4) | 61 (72.6) | 0.024 |
| Primary tumor sites, n (%) |  |  | 0.611 |
| Stomach | 18 (42.9) | 31 (36.9) |  |
| Small intestine | 20 (47.6) | 40 (47.6) |  |
| Colon and rectum | 3 (7.1) | 6 (7.1) |  |
| Others^a^ | 1 (2.4) | 7 (8.3) |  |
| Disease status, n (%) |  |  | 0.697 |
| Initial metastatic GISTs | 15 (35.7) | 33 (39.3) |  |
| Recurrent GISTs | 27 (64.3) | 51 (60.7) |  |
| Median diameter of the largest lesions at the start of imatinib treatment (mm)^b^ | 34.5 (0–130) | 59 (0–170) | 0.058 |
| Pre-imatinib surgery, n (%) | 15 (35.7) | 25 (29.8) | 0.499 |
| No evaluable lesions on CT scan after initial cytoreductive surgery, n (%) | 4 (9.5) | 4 (4.8) | 0.301 |
| Sites of metastasis, n (%) |  |  |  |
| Liver | 23 (54.8) | 50 (59.5) | 0.610 |
| Lung | 0 (0.0) | 1 (1.2) | 0.478 |
| Peritoneum | 19 (45.2) | 40 (47.6) | 0.801 |
| Liver and Peritoneum | 7 (16.7) | 19 (22.6) | 0.436 |
| Genotype of primary tumor^c^, n (%) |  |  | 0.486 |
| KIT exon 11 mutation | 30 (76.9) | 50 (66.7) |  |
| Non-KIT exon 11 mutation | 7 (17.9) | 21 (28.0) |  |
| Surgical resection in RD with TKIs | 16 (38.1) | 15 (17.9) | 0.013 |

a: Esophagus (n = 2), and omentum and peritoneum (n = 6)

b: Not available data (n = 1): no computed tomography (CT) scan

c: available specimens analyzed (n = 108)

Abbreviations: GISTs, gastrointestinal stromal tumors; RD, responsive disease
